# Comprehensive analysis of epidemiological and clinical features of Oropouche virus infection (1975 to 2025): a systematic review

**DOI:** 10.1016/j.nmni.2026.101725

**Published:** 2026-02-17

**Authors:** Xin Wang, Yibo Ding, Amaro Nunes Duarte-Neto, Jaffar A. Al-Tawfiq, Wenshi Wang, Qiuwei Pan, Jiajing Li

**Affiliations:** a Department of Gastroenterology and Hepatology, Erasmus MC-University Medical Center, Rotterdam, the Netherlands; b Department of Pathogen Biology and Immunology, Jiangsu Key Laboratory of Immunity and Metabolism, Jiangsu International Laboratory of Immunity and Metabolism, Xuzhou Medical University, Xuzhou, China; c Departamento de Patologia, Faculdade de Medicina, Universidade de São Paulo, São Paulo, SP, Brazil; d Infectious Disease Unit, Specialty Internal Medicine, Johns Hopkins Aramco Healthcare, Dhahran, Saudi Arabia; e Division of Infectious Diseases, Indiana University School of Medicine, Indianapolis, IN, USA; f Division of Infectious Diseases, Johns Hopkins University, Baltimore, MD, USA; g Accreditation and Infection Control Division, Quality and Patient Safety Department, Johns Hopkins Aramco Healthcare, Dhahran, Saudi Arabia

**Keywords:** Oropouche virus, Oropouche fever, Systematic review, Meta-analysis, Arbovirus, Clinical manifestations, Vertical transmission

## Abstract

**Background:**

Oropouche virus (OROV) has re-emerged since late 2023 with expanding urban transmission in Latin America. OROV causes an acute febrile illness with symptoms overlapping other arboviral infections, complicating diagnosis. Recent reports of fatalities and suspected vertical transmission have raised concerns regarding disease severity. This study aims to provide a comprehensive analysis of epidemiological and clinical features of OROV infection.

**Methods:**

We conducted a PRISMA-compliant systematic review by searching major bibliographic databases and preprint servers. Observational studies reporting OROV infection in febrile patients or the general population were included, and pooled prevalence estimates were calculated.

**Results:**

59 studies were included. The pooled seroprevalence of anti-OROV antibodies in the general population was 7% (95% CI, 0–21%). Among febrile patients, pooled prevalence was 16% (95% CI, 10–24%) by virus detection and 24% (95% CI, 13–36%) by serology. Sex distribution was comparable (male 51% vs female 49%), while infections were most common among individuals aged 20–39 years (38%; 95% CI, 32–43%). The most frequent symptoms were fever (94%; 95% CI, 90–98%), headache (87%; 95% CI, 84–91%), and myalgia (73%; 95% CI, 67–80%), with gastrointestinal and ocular manifestations also common. Compilation of reported fatal cases showed a rapidly progressive clinical course. 17 pregnancy-associated cases suggested possible vertical transmission with heterogeneous outcomes, including congenital abnormalities and fetal loss.

**Conclusions:**

OROV contributes to acute febrile illness in Latin America and presents as a multisystem disease. Emerging reports of fatal outcomes and pregnancy-associated adverse events warrant heightened clinical awareness, improved diagnostics, and strengthened surveillance.

## 1 Introduction

Oropouche virus (OROV) is a re-emerging arbovirus historically endemic to Central and South America, particularly in the Amazon basin [1]. Since late 2023, OROV has shown broader geographic expansion, with an increased at-risk population and spread beyond its endemic region. From January 2024 to December 2025, over 29,000 cases have been reported in Latin America [2], and travel-associated cases have been identified in the United States and Europe [[3], [4], [5]].

Oropouche fever has long been regarded as a mild and self-limiting illness. Most patients present with influenza-like symptoms, such as fever, headache and body pain. These non-specific clinical features highly overlap with symptoms of other arboviral infections, including Dengue and Zika [6,7]. The overlapping clinical manifestations shared by arboviruses complicate differential diagnosis and may contribute to underestimation of OROV circulation.

Although most cases are mild, severe complications have been reported in a small proportion of patients [8]. Notably, fatal cases and vertical transmission as well as associated stillbirths have been reported during the current outbreak [9], although the causative relations remain to be verified. These reports challenge the long-standing perception of OROV infection as benign and raise concerns that severe outcomes may be under-recognized. Summarizing and investigating these reported cases can increase awareness of these severe outcomes of Oropouche fever and provide evidence for identifying potential risk factors.

Despite increasing case numbers, critical knowledge gaps remain in understanding the epidemiology and clinical course of OROV infection. To better manage the ongoing outbreak and prepare for emerging epidemic threats, this study aimed to systematically analyze the epidemiological and clinical features of OROV infection. Further, reported fatal cases and vertical transmissions were summarized.

## 2 Methods

### 2.1 Search strategy

This systematic review and meta-analysis followed the Preferred Reporting Items for Systematic Reviews and Meta-analyses guidelines (Supplemental 2) [10]. The protocol for this study was registered on PROSPERO (International Prospective Register of Systematic Reviews, CRD42024578982). A comprehensive systematic search was conducted across multiple databases: Embase, Medline ALL, Web of Science Core Collection, Web of Science Preprint Citation Index, Cochrane Central Register of Controlled Trials, Global Index Medicus, bioRxiv and medRxiv and Google Scholar. We searched, and further updated for published studies without language restrictions from inception to January 16th, 2026, using an exhaustive set of search terms related to “oropouche”. The full search strategies and selection criteria are detailed in the Supplemental Methods. Two investigators (Xin Wang and Jiajing Li) independently screened the titles and abstracts of all identified citations and reviewed the full-text manuscripts of potentially relevant articles. Disagreements were resolved by consensus and a third reviewer (Qiuwei Pan) if necessary. As our study utilized exclusively published data from existing studies, no additional ethical approval or participant consent was required.

### 2.2 Inclusion and exclusion criteria

This systematic review was structured according to a PEO framework.

The population (P) included febrile patients and general population. Febrile patients were defined as individuals enrolled because of acute febrile illness, whereas general population referred to community-based samples not recruited on the basis of acute febrile illness.

The exposure (E) was OROV infection as defined in each original study, provided that case ascertainment was based on at least one laboratory diagnostic method (virus detection and/or serology). Accepted diagnostic methods include virus detection (reverse transcription-polymerase chain reaction (RT-PCR) and virus isolation), and antibody detection (OROV-specific IgM-enzyme-linked immunosorbent assay (ELISA), IgG-ELISA, hemagglutination inhibition assay (HI) and plaque reduction neutralization test (PRNT)).

The primary outcomes (O) were the prevalence of OROV infection. Secondary outcomes included the spectrum of clinical manifestations, reported fatal cases and pregnancy-associated outcomes.

Studies without confirmed cases of OROV infection were excluded.

### 2.3 Quality assessment and data extraction

Risk of bias of included articles was assessed independently in duplicate, using the Joanna Briggs Institute (JBI) Critical Appraisal Checklist [11]. Studies were not excluded on the basis of their quality score to increase transparency and to ensure all available evidence in this area was reported. Two investigators (Xin Wang and Jiajing Li) extracted data from each included study based on a standardized form. Study characteristics included first author, country, study period, and study design. Study population data contained the total sample size, the number of infected patients, sex, age and diagnostic methods. Clinical information was collected on symptoms. When duplicate data were identified, we excluded the duplicates with the smallest sample size or incomplete data. Details of data collection are provided in the Supplemental Methods.

### 2.4 Statistical analysis

All statistical analyses were performed by STATA 18.0, with a *P* value of 0.05 or less considered statistically significant.

Random-effects meta-analyses were conducted to generate pooled prevalence estimates with corresponding 95% confidence intervals (CI). 95% prediction intervals (PI) were calculated to reflect the expected range of effects in future similar settings. Between-study variance (τ^2^) was estimated using the restricted maximum likelihood (REML) method, and the Knapp–Hartung adjustment was applied to the standard errors and confidence intervals of pooled estimates. Random-effects models were selected a priori to account for the expected heterogeneity arising from differences in study populations, designs, and diagnostic approaches. For pooled proportions, a Freeman–Tukey double arcsine transformation was applied to stabilize variances, and study-level confidence intervals were calculated using the Wilson method. Heterogeneity between studies was assessed using Cochrane Q and *I*^2^ statistic, with an *I*^2^ of at least 50% considered to be substantial heterogeneity [12]. To explore potential sources of heterogeneity, we conducted random-effects meta-regression (REML with Knapp–Hartung adjustment), and we additionally performed leave-one-out analyses to evaluate the influence of individual studies. Publication bias was assessed using funnel plots when at least 10 studies were available, and Egger's test was performed regardless of the number of included studies [12].

## 3 Results

### 3.1 Summary of included studies

**Fig. 1:**
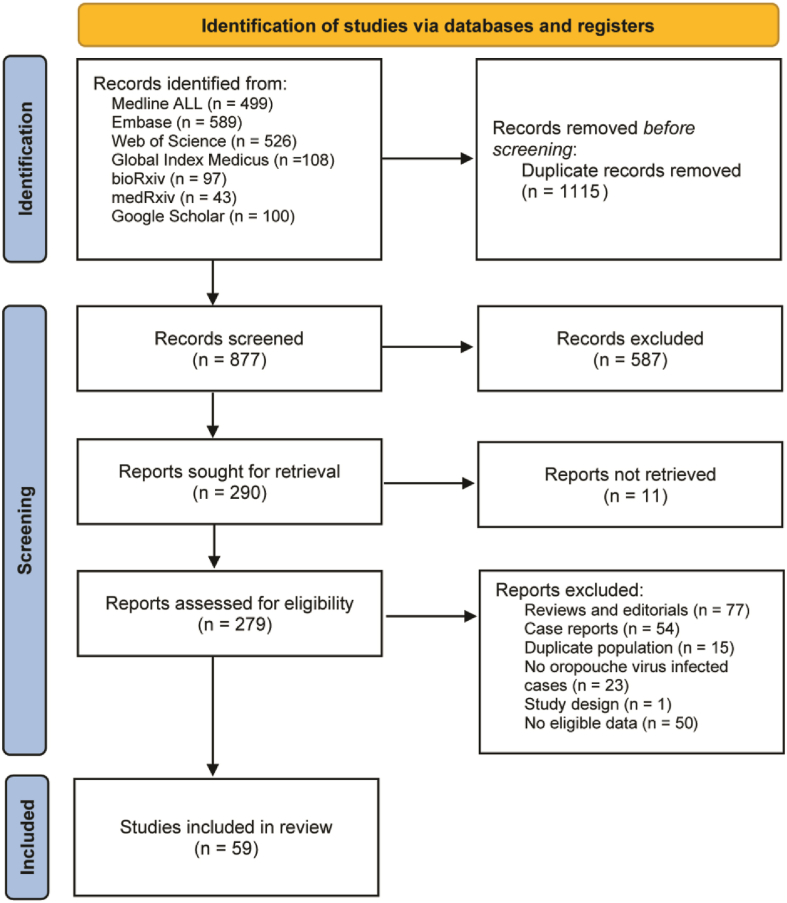
Flow diagram for study selection.

The literature search identified a total of 1992 records. Following the removal of 1115 duplicate entries, 877 unique records remained for screening. Of these, 290 studies were deemed potentially relevant after title and abstract evaluation and were subsequently retrieved for full-text assessment. After full-text review, 220 articles were excluded, resulting in 59 studies being included in the final meta-analysis (Fig. 1, Supplement 1). The key characteristics of the included studies are presented in Supplementary Table S1. Risk of bias in the included studies was assessed using the JBI critical appraisal tool, with domain-level assessments presented in Supplementary Table S2.

### 3.2 OROV prevalence

**Fig. 2:**
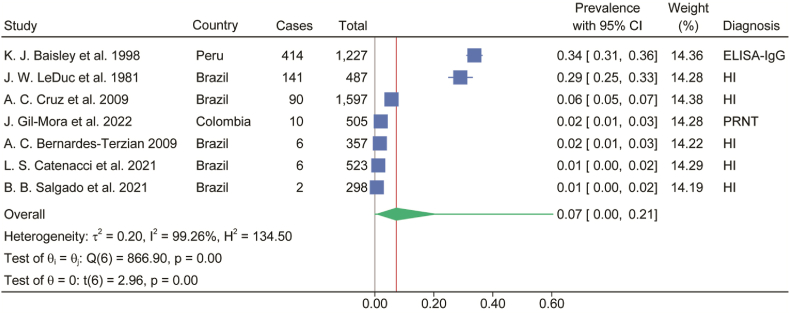
Estimated seroprevalence of anti-OROV antibody positivity in general population where outbreaks occurred. Blue symbol and line represent estimated prevalence of each study with 95% CI. Green symbol represents estimated prevalence with 95% CI. Green line represents 95% prediction interval. ELISA, enzyme-linked immunosorbent assay; HI, hemagglutination inhibiting antibody against the Be An 19991 strain of OROV; PRNT, plaque-reduction neutralization test.

**Fig. 3:**
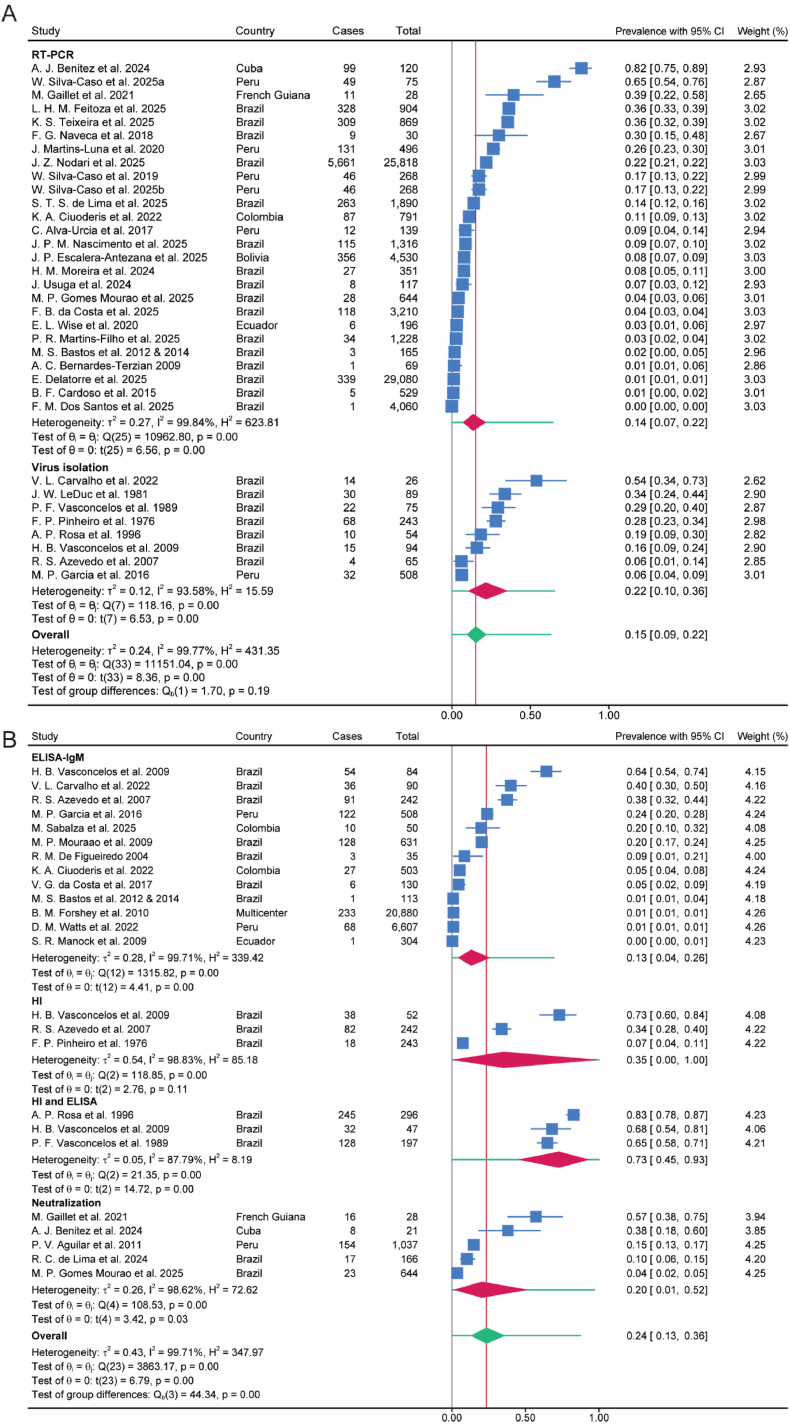
The frequency of OROV infection in patients with acute febrile illness based on detecting virus (A) and antibody (B) in serology tests. Blue symbol and line represent estimated prevalence of each study with 95% CI. Red symbol represents estimated prevalence of subgroup with 95% CI. Green symbol represents estimated prevalence with 95% CI. Green line represents 95% prediction interval. RT-PCR, reverse transcription polymerase chain reaction; ELISA, enzyme-linked immunosorbent assay; HI, Hemagglutination inhibiting antibody against the Be An 19991 strain of OROV.

In regions with reported OROV outbreaks, the estimated seroprevalence of anti-OROV antibody positivity in the general population was 7% (95% CI, 0%-21%) (Fig. 2). Among the included febrile patients, the pooled prevalence of OROV infection was 16% (95% CI, 10%-24%) based on viral detection by virus isolation or RT-PCR detecting the viral genome (Fig. 3A). When assessed by serological evidence of antibodies, the prevalence was 24% (95% CI, 13%-36%) (Fig. 3B).

Between-study heterogeneity was substantial. To explore potential sources of heterogeneity, random-effects meta-regression was performed (where ≥10 studies were available) using study period, diagnostic method, and sample size as moderators. Larger sample size was consistently associated with lower prevalence estimates. In virus-confirmed studies, studies initiated during 2000–2009 showed lower prevalence compared with the post-2020 reference period after adjustment, whereas no consistent association with study period was observed in antibody-confirmed studies (Fig. S2A and S2B). However, these moderators explained only part of the between-study variance, and considerable residual heterogeneity remained.

Leave-one-out analysis demonstrated that the overall pooled prevalence remained generally stable when any single study was omitted (Figs. S1A, S2C, S2D). However, in population-based surveillance studies, results were more sensitive to the exclusion of pre-2000 studies. Excluding the two pre-2000 studies yielded a pooled prevalence of 0.02 (95% CI 0.01–0.04) (Fig. S1B).

Funnel plot asymmetry together with a significant Egger's test suggested possible publication bias (Supplementary Fig. S2E and S2F).

### 3.3 Age and sex characteristics of OROV patients

**Fig. 4:**
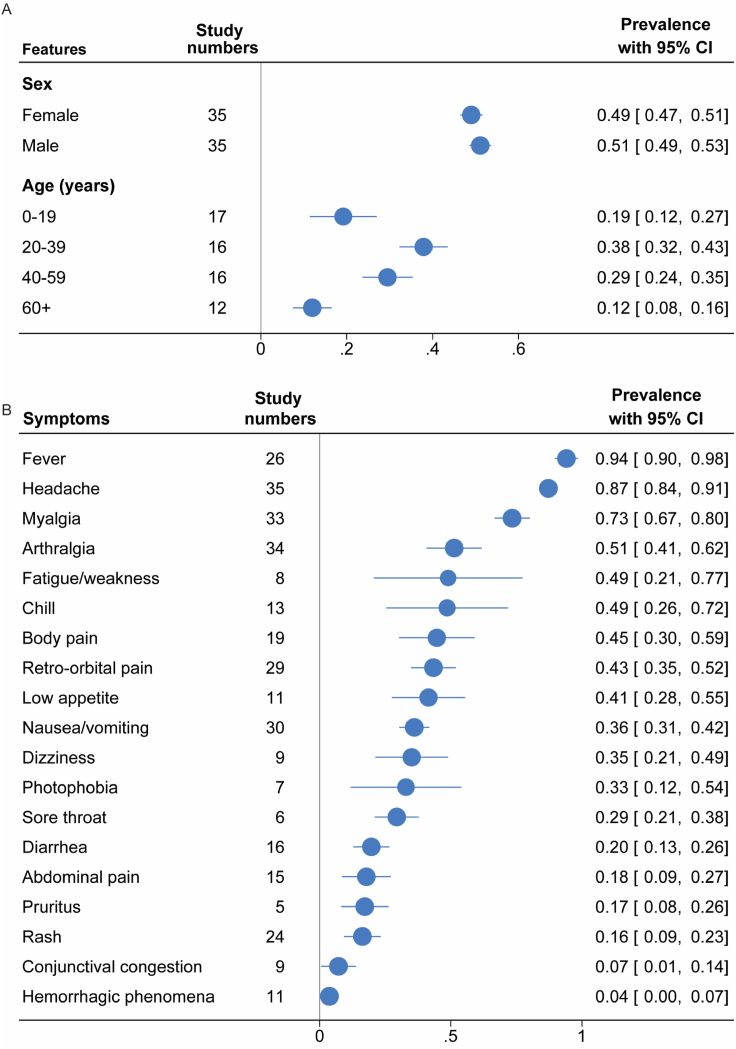
(A) Age and sex distribution in OROV infected patients. (B) Clinical signs and symptoms of OROV infected patients. Blue symbol and line represent estimated prevalence with 95% CI.

Overall, the sex distribution among infected individuals was balanced (male 51%, 95% CI 49%–53%; female 49%, 95% CI 47%–51%) (Fig. 4A and Fig. S3). Age distribution varied across studies. Among the infected patients, the largest proportion was in the 20-39 years age group (38%; 95% CI, 32%-43%), followed by the 40-59 years (29%; 95% CI, 24%-35%), 0-19 years (19%; 95% CI, 12%-27%), and ≥60 years (12%; 95% CI, 8%-16%) (Fig. 4A and Fig. S4).

### 3.4 Clinical manifestations in OROV patients

To characterize the clinical presentation of OROV infection, we synthesized available evidence on the spectrum of reported symptoms (Fig. 4B and Fig. S5). The common clinical symptoms of OROV infection include fever (94%; 95% CI, 90%-98%), headache (87%; 95% CI, 84%-91%), myalgia (73%; 95% CI, 67%-80%), chills (49%; 95% CI, 26%-72%), fatigue or weakness (49%; 95% CI, 21%-77%), dizziness (35%; 95% CI, 21%-49%), body pain (45%; 95% CI, 30%-59%) and arthralgia (51%; 95% CI, 41%-62%). Beyond general symptoms, clinical manifestations were observed across multiple organ systems. Evidence of gastrointestinal manifestations is indicated by nausea or vomiting (36%; 95% CI, 31%-42%), low appetite (41%; 95% CI, 28%-55%), abdominal pain (18%; 95% CI, 9%-27%) and diarrhea (20%; 95% CI, 13%-26%). Ocular involvement is indicated by retro-orbital pain (43%; 95% CI, 35%-52%), photophobia (33%; 95% CI, 12%-54%) and conjunctival congestion (7%; 95% CI, 1%-14%). Skin-related symptoms, including rash (16%; 95% CI, 9%-23%) and pruritus (17%; 95% CI, 8%-26%), were reported in OROV-infected patients. Other symptoms and manifestations include sore throat (29%; 95% CI, 21%-38%) and hemorrhagic phenomena (4%; 95% CI, 0%-7%).

### 3.5 Severe outcomes of OROV infection

Although OROV infection is self-limiting in most patients, fatal cases were reported during the 2024 outbreak. We summarized three reported fatal cases with detailed information (Table 1). All the fatal cases showed a rapidly progressive course, with death occurring within 4–5 days of symptom onset. Two cases involved young women without underlying conditions, who developed prominent gastrointestinal symptoms with coagulation abnormalities and renal dysfunction [13]. The third case occurred in an older woman with hypertension and obesity and progressed rapidly to acute respiratory distress, severe coagulopathy, and multiorgan failure [14].

**Table 1:** Clinical characteristics of reported fatal cases of Oropouche virus infection.

Case number	Year	Sex	Age	Underlying conditions	Symptom	Complications	OROV infection	Symptom onset to death
Case 1	2024	Female	24	History of miscarriage	Fever, headache, retroorbital pain, myalgia, severe abdominal pain, diarrhea, nausea, blurred vision, and vomiting	Ocular edema, psychomotor agitation, hypotension, desaturation, liver injury and renal dysfunction	RT-PCR positive	4 days
Case 2	2024	Female	21	No	Fever, myalgia, headache, retroorbital pain, pain in the lower limbs, asthenia, joint pain, weakness, drowsiness, and vomiting	Thrombocytopenia, prolongation of clotting and bleeding time, and renal dysfunction	RT-PCR positive	5 days
Case 3	2024	Female	61	Hypertension and obesity	Dyspnea and fever	Acute respiratory distress, leukocytosis with neutrophilia, thrombocytopenia, severe coagulopathy and multiorgan failure	RT-PCR positive	4 days

### 3.6 Vertical transmission

In addition to fatal outcomes, vertical transmission has also been reported in OROV infection. We further summarized reported cases of vertical transmission of OROV (Table 2). A total of 17 pregnancy-associated cases from six reports published between 2016 and 2025 were included [[15], [16], [17], [18], [19], [20]], most of which occurred during the 2024 outbreak. Among these, 14 cases (Cases 1–14) had laboratory evidence of OROV infection in both the pregnant individual and the fetus or neonate, based on laboratory confirmation, providing direct evidence consistent with confirmed vertical transmission. The remaining three cases (Cases 15–17) were associated with miscarriage or stillbirth and had laboratory confirmation of maternal OROV infection only; no fetal or placental testing results were available in these cases. These cases are therefore considered suggestive of possible vertical transmission. Maternal infection was reported across all trimesters, with common symptoms including fever, rash, headache, retro-orbital pain, and myalgia. Perinatal outcomes were heterogeneous, ranging from normal outcomes to severe adverse events, including microcephaly and other congenital abnormalities, early neonatal death, miscarriage, and intrauterine fetal demise.

**Table 2:** Reported cases of vertical transmission associated with Oropouche virus infection.

Case number	Year	Age of pregnancy	Underlying conditions	Onset of symptom	Symptoms in pregnancy	OROV in neonates	Symptoms in neonates	Outcome of neonates
case1	2016	33	HIV	First trimester	Rash	ELISA-IgM positive	Loose redundant and folded skin on the head, and bilateral equinovarus feet	Microcephaly; died at age 2 days
case2	2016	NA	ELISA-IgG positive for toxoplasmosis	NA	Asymptomatic	ELISA-IgM positive	Microcephaly	Microcephaly; alive
case3	2018	16	No	First trimester	Fever	ELISA-IgM positive; HI positive	Microcephaly, seizures, tremors, and muscle twitching	Microcephaly; alive
case4	2024	22	ELISA-IgG positive for rubella, cytomegalovirus, and herpes	Fifth month	Fever, rash, headache, retro-orbital pain, and myalgia	ELISA-IgM positive; HI positive	Skull collapse with loose redundant and folded skin on the head	Microcephaly; alive
case5	2024	37	ELISA-IgG positive for toxoplasmosis, rubella, and cytomegalovirus	Second month	Fever, rash, headache, retro-orbital pain, myalgia, and vomiting	ELISA-IgM positive; HI positive	Subcutaneous edema (hydrops), craniofacial disproportion, small skull, overlapping cranial sutures, short neck, multiple arthrogryposis, camptodactyly, and right club foot	Microcephaly; alive
case6	2024	33	No	Second month	Fever, headache, rash, retro-orbital pain, and myalgia	ELISA-IgM positive; HI positive; RT-PCR positive	Loose redundant and folded skin on the head, multiple arthrogryposis, camptodactyly, and bilateral cryptorchidism	Microcephaly; died at age 47 days
case7	2024	32	No	38th week	Fever, severe headache, dizziness, nausea, skin rash, muscle pain, joint pain, and lower back pain	ELISA-IgM positive	Fever	Normal
case8	2024	21	No	29th week	Fever, severe headache, and muscle pain	RT-PCR positive, lowest Ct in brain	NA	Intrauterine Fetal Demise
case9	2024	NA	No	30th week	Fever, headache, rash, and epigastric pain	RT-PCR positive	NA	Intrauterine Fetal Demise
case10	2025	20	No	39th week	Fever, headache, myalgia, back pain	RT-PCR positive	Recurrent fever and agitation	Normal
case11	2024	40	Gestational diabetes mellitus	30th week	Fever, chills, myalgia, headache, vaginal bleeding and dark vaginal discharge	RT-PCR positive	NA	Intrauterine Fetal Demise
case12	2024	22	No	21st week	Fever, rash, headache, myalgia, and retro-orbital pain	ELISA-IgM positive	NA	Microcephaly; alive
case13	2024	NA	NA	7th week	Fever, myalgia, headache, nausea, retro-ocular pain	RT-PCR positive	Asymptomatic	Dysgenesis of the corpus callosum
case14	2024	NA	NA	38th week	Fever, headache, myalgia	RT-PCR positive	Fever, exanthema, and agitation	Normal
case15	2024	34	Diabetes and grade 1 obesity	6th week	Fever, severe headache, dizziness, nausea, skin rash, muscle pain, and lower back pain	NA	NA	Miscarriage
case16	2024	43	No	38th week	Fever, headache, bilateral mastitis	NA	NA	Intrauterine Fetal Demise
case17	2024	NA	NA	7th week	Fever, headache, myalgia, back pain, retro-ocular pain, nausea, and arthralgia	NA	NA	Spontaneous abortion at 8 weeks of pregnancy

Note: NA: not available; HI: hemagglutination inhibition; RT-PCR, reverse transcription polymerase chain reaction; ELISA, enzyme-linked immunosorbent assay.

## 4 Discussion

This study comprehensively estimated the prevalence and clinical features of OROV infection through a systematic review and meta-analysis of studies conducted mainly in Latin America, including both recent and historical studies.

Our pooled analyses indicate that OROV infection represents a substantial burden in outbreak-affected regions. In the general population, pooled seroprevalence was 7%, suggesting limited cumulative exposure in most settings, although substantial between-study heterogeneity was observed. Notably, two studies reporting markedly higher seroprevalence were conducted in contexts of intensified transmission. One was performed following a documented outbreak and the other in an established Amazonian endemic hotspot with sustained viral circulation, where seropositivity reflects amplified cumulative exposure rather than background transmission. In contrast, other studies reported substantially lower seroprevalence (1–6%), representing baseline circulation levels. Sensitivity analyses showed that the pooled estimate was not driven by any single study, and decreased to approximately 2% when the two high-transmission studies were excluded, consistent with background exposure. Together, these findings suggest that the observed heterogeneity primarily reflects a gradient of transmission intensity across ecological contexts rather than methodological inconsistency.

Among febrile patients in endemic and epidemic settings, OROV accounted for a large proportion of infections. These findings indicate that OROV is not a rare pathogen in endemic and epidemic settings. However, potential publication bias and substantial residual heterogeneity indicate that pooled estimates should be interpreted cautiously. Meta-regression suggested that sample size and study period contributed to between-study variability, but explained only a limited proportion of the heterogeneity. The inclusion of studies spanning nearly four decades adds variability related to evolving diagnostic technologies and surveillance practices. Earlier serological studies from highly endemic regions may reflect cumulative exposure rather than point prevalence, which can yield higher seroprevalence estimates.

Funnel plot asymmetry and significant Egger's tests indicated potential small-study effects/publication bias, suggesting that the pooled prevalence may be distorted by selective reporting. However, because prevalence meta-analyses are sensitive to heterogeneity and variance instability, these signals should be interpreted cautiously because asymmetry may also arise from genuine differences in study settings, sampling frames, and diagnostic approaches. Furthermore, the direction and magnitude of bias cannot be certainly determined.

Sex-stratified analysis showed no meaningful difference in infection prevalence between males and females, suggesting that sex-specific biological factors play a limited role in susceptibility at the population level. Intriguingly, our finding suggests that the young adult group is highly susceptible to the infection. This pattern likely reflects exposure-related factors rather than intrinsic biological susceptibility. Young adults may experience higher vector exposure due to occupational activities, mobility, and social behavior. It may also reflect that older age groups have accumulated higher levels of anti-OROV antibodies due to repeated or long-term exposure [21], which could partially protect them against acute infection. In contrast, younger individuals, with lower cumulative exposure and seroprevalence, may therefore be more frequently represented among acute OROV cases.

Clinically, the pooled estimates confirm that OROV infection can affect multiple organs. Fever, headache, and myalgia were the most frequently reported symptoms, consistent with prior descriptions of Oropouche fever. Gastrointestinal, ocular and skin manifestations overlap with those of other co-circulating arboviruses [[22], [23], [24]]. Consequently, in the absence of laboratory confirmation, distinguishing OROV infection from other arboviral infections remains challenging in clinical practice.

Beyond the clinical manifestations, our findings draw attention to the potential of severe outcomes associated with OROV infection. No OROV-related fatal cases were documented before the 2024 outbreak. Since late 2023, fatal cases were confirmed with OROV infection in Brazil and detailed information of three cases were available. By synthesizing these reports, we observed that fatal outcomes can occur in young patients without underlying conditions. In these cases, OROV infection exhibited a rapidly progressive course, leading to severe complications with multisystem involvement.

In addition, our synthesis of pregnancy-associated OROV infections highlights growing concern regarding vertical transmission. Across the summarized cases, maternal infection occurred in all trimesters. Reported perinatal outcomes were heterogeneous, ranging from normal outcomes to severe adverse events. However, the available evidence is derived primarily from case reports and small case series, and alternative etiologies were not systematically excluded in most instances. While *in vitro* studies have demonstrated that OROV can infect placental explants [25], the clinical mechanisms and risk factors underlying vertical transmission remain incompletely understood. Similar concerns have been raised for other arbovirus such as Zika virus [26,27]. These findings highlight the need for closer obstetric follow-up in pregnancy infected with arboviruses, given the risk of adverse outcomes.

This study has several limitations. First, although we included all available datasets, the overall number of eligible studies remains limited, and we were unable to conduct a robust comparison between historical and current outbreaks. Second, substantial heterogeneity persisted across prevalence estimates, sample size and study period contributed to variability, but a large proportion of residual heterogeneity remained unexplained. Third, diagnostic approaches and case definitions were not fully uniform across studies, especially across different study periods, and this may have contributed to between-study variability. Some studies were conducted in specific clinical or outbreak settings, which may affect the generalizability of pooled estimates. Finally, funnel plot asymmetry and Egger's test suggested possible publication bias, indicating that pooled prevalence estimates should be interpreted with caution.

In summary, our findings suggest that OROV infection should no longer be regarded as a neglected or mild arboviral disease. Instead, OROV represents an emerging epidemic threat with important diagnostic, clinical, and obstetric implications. The substantial heterogeneity observed across studies highlights persistent gaps in surveillance, laboratory capacity, and standardized reporting, underscoring the need for capacity building in laboratory diagnostics and epidemiological monitoring. Future efforts should prioritize prospective and standardized studies. Increased clinical awareness, improved access to reliable diagnostics, and strengthened surveillance systems are particularly needed in settings experiencing concurrent arboviral outbreaks.

## CRediT authorship contribution statement

**Xin Wang:** Writing – original draft, Investigation, Formal analysis, Data curation. **Yibo Ding:** Writing – review & editing, Formal analysis, Data curation. **Amaro Nunes Duarte-Neto:** Writing – review & editing, Data curation. **Jaffar A. Al-Tawfiq:** Writing – review & editing, Investigation. **Wenshi Wang:** Writing – review & editing, Investigation. **Qiuwei Pan:** Writing – original draft, Supervision, Project administration, Data curation, Conceptualization. **Jiajing Li:** Writing – original draft, Visualization, Methodology, Investigation, Formal analysis, Data curation, Conceptualization.

## Funding

None.

## Declaration of competing interest

The authors declare that they have no known competing financial interests or personal relationships that could have appeared to influence the work reported in this paper.
