# Supplementary material for: Comprehensive analysis of epidemiological and clinical features of Oropouche virus infection (1975 to 2025): a systematic review
Source: New Microbes New Infect. 2026 Feb 17;70:101725. doi: 10.1016/j.nmni.2026.101725 (PMC12936821; doi:10.1016/j.nmni.2026.101725)
Supplement: Multimedia component 1 [file mmc1.pdf]

Supplement 1

Methods: Search strategy, selection criteria and data collection

This systematic review and meta-analysis followed the Preferred Reporting Items for Systematic Reviews and Meta-analyses (PRISMA) guidelines (Supplement 2). The study protocol was registered with PROSPERO (CRD42024578982) and available online.

Search strategy

An exhaustive search strategy was developed by an experienced information specialist (M.F.M.) in cooperation with one of the investigators (J.L.). The search was developed in Embase.com, optimized for sensitivity and then translated to other databases following the method as described by Bramer et al.[1] The search strategies for Medline and Embase used relevant thesaurus terms from Medical Subject Headings (MeSH) and Emtree respectively. In both databases, terms were searched in titles, abstracts and author keywords. Terms were combined with Boolean operators AND and OR and proximity operators were used to combine terms into phrases. The references were imported into EndNote and duplicates were removed by an experienced information specialist (M.F.M.) using the method as described by Bramer et al.[2]

Database searched	Platform	Years of coverage	Records	Records after duplicates removed
Medline ALL	Ovid	1946 - Present	499	497
Embase	Embase.com	1971 - Present	589	119
Web of Science Core Collection*	Web of Knowledge	1975 - Present	526	73
Web of Science Preprint Citation Index	Web of Knowledge		30	5
Cochrane Central Register of Controlled Trials*	Wiley	1992 - Present	0	0
Global Index Medicus	www.globalindexmedicus.net		108	72
bioRxiv	www.biorxiv.org		97	78
medRxiv	www.medrxiv.org		43	26
Additional Search Engines: Google Scholar** (100 top-ranked)			100	7

**Total**	**1992**	**877**

*Science Citation Index Expanded (1975-present) ; Social Sciences Citation Index (1975-present) ; Arts & Humanities Citation Index (1975-present) ; Conference Proceedings Citation Index- Science (1990-present) ; Conference Proceedings Citation Index- Social Science & Humanities (1990-present) ; Emerging Sources Citation Index (2005-present)

**Google Scholar was searched via "Publish or Perish" to download the results in EndNote.

No other database limits were used than those specified in the search strategies

Embase
('Oropouche orthobunyavirus'/exp OR (oropouche* OR OROV):ab,ti,kw)
Medline
(Oropouche orthobunyavirus.rs. OR (oropouche* OR OROV).ab,ti,kf.)
Cochrane
((oropouche* OR OROV):ab,ti,kw)
Web of Science
TS=(oropouche* OR OROV)
Global Index Medicus
oropouche* OR OROV
bioRxiv
oropouche OR OROV
medRxiv
oropouche OR OROV
Google Scholar
oropouche|orov

References

[1] Bramer WM, de Jonge GB, Rethlefsen ML, Mast F, Kleijnen J. A systematic approach to searching: an efficient and complete method to develop literature searches. J Med Libr Assoc 2018;106(4):531-41.

[2] Bramer WM, Giustini D, de Jonge GB, Holland L, Bekhuis T. De-duplication of database search results for systematic reviews in EndNote. J Med Libr Assoc 2016;104(3):240-3.
