# Supplementary material for: Comprehensive analysis of epidemiological and clinical features of Oropouche virus infection (1975 to 2025): a systematic review
Source: New Microbes New Infect. 2026 Feb 17;70:101725. doi: 10.1016/j.nmni.2026.101725 (PMC12936821; doi:10.1016/j.nmni.2026.101725)
Supplement: Multimedia component 2 [file mmc2.pdf]

Supplement 2

PRISMA

Section and Topic	Item #	Checklist item	Reported on page #
**TITLE**			
Title	1	Identify the report as a systematic review.	1
**ABSTRACT**			
Structured summary	2	Provide a structured summary including, as applicable: background; objectives; data sources; study eligibility criteria, participants, and interventions; study appraisal and synthesis methods; results; limitations; conclusions and implications of key findings; systematic review registration number.	3
**INTRODUCTION**			
Rationale	3	Describe the rationale for the review in the context of existing knowledge.	5
Objectives	4	Provide an explicit statement of the objective(s) or question(s) the review addresses.	5
**METHODS**			
Eligibility criteria	5	Specify the inclusion and exclusion criteria for the review and how studies were grouped for the syntheses.	6, 7 and Supplement 1
Information sources	6	Specify all databases, registers, websites, organisations, reference lists and other sources searched or consulted to identify studies. Specify the date when each source was last searched or consulted.	6 and Supplement 1
Search strategy	7	Present the full search strategies for all databases, registers and websites, including any filters and limits used.	6 and Supplement 1
Selection process	8	Specify the methods used to decide whether a study met the inclusion criteria of the review, including how many reviewers screened each record and each report retrieved, whether they worked independently, and if applicable, details of automation tools used in the process.	6 and Supplement 1
Data collection process	9	Specify the methods used to collect data from reports, including how many reviewers collected data from each report, whether they worked independently, any processes for obtaining or confirming data from study investigators, and if applicable, details of automation tools used in the process.	6 and Supplement 1
Data items	10a	List and define all outcomes for which data were sought. Specify whether all results that were compatible with each outcome domain in each study were sought (e.g. for all measures, time points, analyses), and if not, the methods used to decide which results to collect.	Supplement 1<br>and Table S1
	10b	List and define all other variables for which data were sought (e.g. participant and intervention characteristics, funding sources). Describe any assumptions made about any missing or unclear information.	Supplement 1 and Table S1
Study risk of bias assessment	11	Specify the methods used to assess risk of bias in the included studies, including details of the tool(s) used, how many reviewers assessed each study and whether they worked independently, and if applicable, details of automation tools used in the process.	7
Effect measures	12	Specify for each outcome the effect measure(s) (e.g. risk ratio, mean difference) used in the synthesis or presentation of results.	7-8
Synthesis methods	13a	Describe the processes used to decide which studies were eligible for each synthesis (e.g. tabulating the study intervention characteristics and comparing against the planned groups for each synthesis (item #5)).	7-8
	13b	Describe any methods required to prepare the data for presentation or synthesis, such as handling of missing summary statistics, or data conversions.	
	13c	Describe any methods used to tabulate or visually display results of individual studies and syntheses.	
	13d	Describe any methods used to synthesize results and provide a rationale for the choice(s). If meta-analysis was performed, describe the model(s), method(s) to identify	7-8

Section and Topic	Item #	Checklist item	Reported on page #
		the presence and extent of statistical heterogeneity, and software package(s) used.	
	13e	Describe any methods used to explore possible causes of heterogeneity among study results (e.g. subgroup analysis, meta-regression).	7-8
	13f	Describe any sensitivity analyses conducted to assess robustness of the synthesized results.	
Reporting bias assessment	14	Describe any methods used to assess risk of bias due to missing results in a synthesis (arising from reporting biases).	
Certainty assessment	15	Describe any methods used to assess certainty (or confidence) in the body of evidence for an outcome.	7-8
**RESULTS**			
Study selection	16a	Describe the results of the search and selection process, from the number of records identified in the search to the number of studies included in the review, ideally using a flow diagram.	Figure 1
	16b	Cite studies that might appear to meet the inclusion criteria, but which were excluded, and explain why they were excluded.	
Study characteristics	17	Cite each included study and present its characteristics.	Table S1
Risk of bias in studies	18	Present assessments of risk of bias for each included study.	Figure S1 and Table S2
Results of individual studies	19	For all outcomes, present, for each study: (a) summary statistics for each group (where appropriate) and (b) an effect estimate and its precision (e.g. confidence/credible interval), ideally using structured tables or plots.	Figure 2,3, and S2-4
Results of syntheses	20a	For each synthesis, briefly summarise the characteristics and risk of bias among contributing studies.	
	20b	Present results of all statistical syntheses conducted. If meta-analysis was done, present for each the summary estimate and its precision (e.g. confidence/credible interval) and measures of statistical heterogeneity. If comparing groups, describe the direction of the effect.	9-10 and Figure 2,3, S1-4
	20c	Present results of all investigations of possible causes of heterogeneity among study results.	Figure S1-2
	20d	Present results of all sensitivity analyses conducted to assess the robustness of the synthesized results.	
Reporting biases	21	Present assessments of risk of bias due to missing results (arising from reporting biases) for each synthesis assessed.	
Certainty of evidence	22	Present assessments of certainty (or confidence) in the body of evidence for each outcome assessed.	Table S1
**DISCUSSION**			
Discussion	23a	Provide a general interpretation of the results in the context of other evidence.	12
	23b	Discuss any limitations of the evidence included in the review.	14-15
	23c	Discuss any limitations of the review processes used.	14-15
	23d	Discuss implications of the results for practice, policy, and future research.	14
**OTHER INFORMATION**			
Registration and protocol	24a	Provide registration information for the review, including register name and registration number, or state that the review was not registered.	6
	24b	Indicate where the review protocol can be accessed, or state that a protocol was not prepared.	
	24c	Describe and explain any amendments to information provided at registration or in the protocol.	
Support	25	Describe sources of financial or non-financial support for the review, and the role of the funders or sponsors in the review.	2

Section and Topic	Item #	Checklist item	Reported on page #
Competing interests	26	Declare any competing interests of review authors.	2
Availability of data, code and other materials	27	Report which of the following are publicly available and where they can be found: template data collection forms; data extracted from included studies; data used for all analyses; analytic code; any other materials used in the review.	Supplement 1 and 2

Table S1 : Characteristics of included studies

Study	Country	Study period	Virus detection	Sample size	Virus-proved cases	Antibody detection	Sample size	Antibody-proved cases	PMID
**In acute febrile illness patients**									
F. P. Pinheiro et al. 1976	Brazil	From February to April 1975	Virus isolation	243	68	Hemagglutination-inhibiting against the Be An 19991 and IgM capture ELISA	243	18	941251
J. W. LeDuc et al. 1981	Brazil	From early July until late September 1978	Virus isolation	89	30				7326505
P. F. Vasconcelos et al. 1989	Brazil	From January 26 to February 1 1988	Virus isolation	75	22	Hemagglutination-inhibiting against the Be An 19991 and IgM capture ELISA	197	128	2516642
A. P. Rosa et al. 1996	Brazil	From November to December 1994	Virus isolation	54	10	Hemagglutination-inhibiting against the Be An 19991 and IgM capture ELISA	296	245	9011877
R. M. De Figueiredo 2004	Brazil	From March 1998 to December 1999				IgM capture ELISA	35	3	15765597
R. S. Azevedo et al. 2007	Brazil	DurIng 2003 and 2004	Virus isolation	65	4	Hemagglutination-inhibiting against the Be An 19991	234	82	17553235
						IgM capture ELISA		91	
A. C. Bernardes-Terzian 2009	Brazil	From March 2004 to October 2006	RT-PCR	69	1				19193295
S. R. Manock et al. 2009	Ecuador	From April 2001 to September 2004				IgM capture ELISA	304	1	19556580
M. P. Mouraao et al. 2009	Brazil	From January 2007 to November 2008				IgM capture ELISA	631	128	19961705
H. B. Vasconcelos et al. 2009	Brazil	From 29 May to 30 June 2006	Virus isolation	94	15	Hemagglutination-inhibiting against the Be An 19991 and IgM capture ELISA	183	124	19117799
B. M. Forshey et al. 2010	Ecuador, Peru, Bolivia, and Paraguay	Between May 2000 and December 2007				IgM capture ELISA	20880	233	20706628
B. F. Cardoso et al. 2015	Brazil	Between October 2011 and July 2012	RT-PCR	529	5				26517653
M. P. Garcia et al. 2016	Peru	Between December 14, 2015 and January 8, 2016	RT-PCR	508	19	IgM capture ELISA	508	122	27656945
			Virus isolation	508	32				
C. Alva-Urcia et al. 2017	Peru	From January to March 2016	RT-PCR	139	12				29136650
V. G. da Costa et al. 2017	Brazil	From 2011 to 2013				IgM EIA-ICC	130	6	28739422

F. G. Naveca et al. 2018	Brazil	Between April and June 2015	RT-PCR	30	9				29623245
W. Silva-Caso et al. 2019	Peru	Between January and July 2016	RT-PCR	268	46				30991139
J. Martins-Luna et al. 2020	Peru	From February to September 2016	RT-PCR	496	131				32041646
E. L. Wise et al. 2020	Ecuador	In 2016	RT-PCR	196	6				31961856
M. Gaillet et al. 2021	French Guiana	From August 11 to October 15 2020	RT-PCR	28	11	Neutralization	28	16	34545800
V. L. Carvalho et al. 2022	Brazil	From January to February 2018	Virus isolation	26	14	IgM capture ELISA	90	36	35405330
K. A. Ciuoderis et al. 2022	Colombia	During 2019-2022	RT-PCR	791	87	IgM capture ELISA	503	27	36239235
D. M. Watts et al. 2022	Peru	From October 1, 1993 to September 30, 1999				IgM ELISA	6607	68	36162442
R. C. de Lima et al. 2024	Brazil	Between August 2014 and May 2015				Neutralization	166	17	38921767
H. M. Moreira et al. 2024	Brazil	From January 2022 to March 2023	RT-PCR	351	27				38323826
P. V. Aguilar et al. 2011	Peru	In 2006				Neutralization	1037	154	21949892
M. S. Bastos et al. 2012 & 2014	Brazil	From 2005 to August 2012	RT-PCR	165	3	IgM capture ELISA	110	3	22492162 24760682
L. H. M. Feitoza et al. 2025	Brazil	From January 2024 to April 2024	RT-PCR	904	328				39622307
J. Usuga et al. 2024	Brazil	January 2024	RT-PCR	117	8				39356574
A. J. Benitez et al. 2024	Cuba	May 2024	RT-PCR	120	99	Neutralization	21	8	39255237
F. B. da Costa et al. 2025	Brazil	From September 2023 to March 2024	RT-PCR	3210	118				40872343
J. Z. Nodari et al. 2025	Brazil	From January 2023 to December 2024	RT-PCR	25818	5661				40819672
F. M. Dos Santos et al. 2025	Brazil	From January 2023 to June 2024	RT-PCR	4060	1				40788114
K. S. Teixeira et al. 2025	Brazil	From January 2024 to July 2024	RT-PCR	869	309				40716624
J. P. Escalera-Antezana et al. 2025	Bolivia	In 2024	RT-PCR	4530	356				40612647
E. Delatorre et al. 2025	Brazil	From March to June 2024	RT-PCR	29080	339				40401583
S. T. S. de Lima et al. 2025	Brazil	From January to December 2024	RT-PCR	1890	263				40072503
W. Silva-Caso et al. 2025a	Peru	In 2016	RT-PCR	75	49				41438624
M. P. Gomes Mourao et al. 2025	Brazil	From January to March of 2024	RT-PCR	644	28	Neutralization	644	23	41042827
J. P. M. Nascimento et al. 2025	Brazil	From April to September 2024	RT-PCR	1316	115				41459755

M. Sabalza et al. 2025	Colombia	From 2015 to 2016				Elisa-IgM	50	10	10.1093/clinchem/hvaf086.271
W. Silva-Caso et al. 2025b	Peru	From November 2015 to July 2016	RT-PCR	268	46				EMBASE: L620731177
P. R. Martins-Filho et al. 2025	Brazil	From April 1 to September 6, 2024	RT-PCR	1228	34				40243799
**In general population**									
J. W. LeDuc et al. 1981	Brazil	In May and June 1979				Hemagglutination-inhibiting against the Be An 19991	567	170	7326505
K. J. Baisley et al. 1998	Peru	During June and September 1996				IgG capture ELISA	1227	414	9840586
A. C. Bernardes-Terzian 2009	Brazil	During March and April 2004				Hemagglutination-inhibiting against the Be An 19991	357	6	19193295
A. C. Cruz et al. 2009	Brazil	Between October 2006 and December 2007				Hemagglutination-inhibiting against the Be An 19991	1597	90	19936489
L. S. Catenacci et al. 2021	Brazil	NA				Hemagglutination-inhibiting against the Be An 19991	523	6	33538403
B. B. Salgado et al. 2021	Brazil	Between January 2014 and December 2015				Hemagglutination-inhibiting against the Be An 19991	298	2	34391467
J. Gil-Mora et al. 2022	Colombia	In 2018				Neutralization	505	10	36375460
**Confirmed OROV cases**									
J. P. Cola et al. 2025	Brazil	From March 20, 2024 to May 30, 2025	RT-PCR		12135				41450792
F. G. Naveca et al. 2024	Brazil	From January 2022 to March 2024	RT-PCR		2272				39293488
M. E. Toledo et al. 2024	Cuba	In May 2024	RT-PCR		20				38972329
E. B. Martins et al. 2025	Brazil	From 20 December 2024, to 8 May 2025	RT-PCR		55				41509678
B. M. Bello-Rodríguez et al. 2025	Cuba	From June to December 2024	RT-PCR		38				40086738
F. C. de Melo Iani et al. 2025	Brazil	From 2022 to 2024	RT-PCR		133				40037296
A. Morrison et al. 2024	USA	From May 2024 to July 2024	RT-PCR		14	Neutralization		8	39236058
E. A. Ramírez-García et al. 2025	Peru	From December 2023 to September 2024	RT-PCR		496				41213150
N. Labiod et al. 2025	Spain	From 1 June to 31 July 2024	RT-PCR		12	Neutralization		1	41104495
O. Castro Peraza et al. 2025	Cuba	2024	RT-PCR		47				10.2139/ssrn.5391646

Note: NA: not available.

Table S2 : Risk of bias assessment of included studies

Study	Question							
	1	2	3	4	5	6	7	8
F. P. Pinheiro et al. 1976	Y	Y	Y	Y	N	N	Y	Y
J. W. LeDuc et al. 1981	Y	Y	Y	Y	Y	N	Y	Y
K. J. Baisley et al. 1998	N	Y	Y	Y	Y	Y	Y	Y
P. F. Vasconcelos et al. 1989	Y	Y	Y	Y	Y	Y	Y	Y
A. P. Rosa et al. 1996	Y	Y	Y	Y	Y	Y	Y	Y
R. M. De Figueiredo 2004	Y	Y	Y	Y	Y	Y	Y	Y
R. S. Azevedo et al. 2007	Y	Y	Y	Y	N	N	Y	Y
A. C. Bernardes-Terzian 2009	Y	Y	Y	Y	N	N	Y	Y
S. R. Manock et al. 2009	Y	Y	Y	Y	Y	Y	Y	Y
M. P. Mouraao et al. 2009	Y	Y	Y	Y	N	N	Y	Y
H. B. Vasconcelos et al. 2009	Y	Y	Y	Y	Y	Y	Y	Y
B. M. Forshey et al. 2010	Y	Y	Y	Y	Y	Y	Y	Y
B. F. Cardoso et al. 2015	Y	Y	Y	Y	Y	N	Y	N
M. P. Garcia et al. 2016	Y	Y	Y	Y	Y	N	Y	N
C. Alva-Urcia et al. 2017	Y	Y	Y	Y	N	N	Y	N
V. G. da Costa et al. 2017	Y	Y	Y	Y	N	N	Y	N
F. G. Naveca et al. 2018	Y	Y	Y	Y	N	N	Y	N
W. Silva-Caso et al. 2019	Y	Y	Y	Y	N	N	Y	N
J. Martins-Luna et al. 2020	Y	Y	Y	Y	N	N	Y	N
E. L. Wise et al. 2020	Y	Y	Y	Y	N	N	Y	Y
M. Gaillet et al. 2021	Y	Y	Y	Y	N	N	Y	Y
V. L. Carvalho et al. 2022	Y	Y	Y	Y	N	N	Y	Y
K. A. Ciuoderis et al. 2022	Y	Y	Y	Y	Y	Y	N	Y
D. M. Watts et al. 2022	Y	Y	N	Y	Y	Y	N	Y
R. C. de Lima et al. 2024	Y	Y	Y	Y	N	N	N	Y
H. M. Moreira et al. 2024	Y	N	Y	Y	N	N	N	Y
P. V. Aguilar et al. 2011	Y	Y	Y	Y	Y	Y	Y	Y
M. S. Bastos et al. 2012	Y	Y	Y	Y	Y	Y	Y	N
M. S. Bastos et al. 2014	Y	Y	Y	Y	Y	Y	Y	Y
L. H. M. Feitoza et al. 2025	Y	Y	N	Y	N	N	Y	Y
F. G. Naveca et al. 2024	Y	Y	Y	Y	Y	Y	Y	Y
J. Usuga et al. 2024	N	Y	Y	Y	Y	N	Y	Y
A. J. Benitez et al. 2024	Y	Y	Y	Y	N	N	Y	Y
A. Morrison et al. 2024	Y	Y	Y	Y	N	N	Y	Y
M. E. Toledo et al. 2024	N	N	Y	Y	N	N	N	N
F. B. da Costa et al. 2025	Y	Y	Y	Y	N	N	N	Y
J. Z. Nodari et al. 2025	Y	Y	Y	Y	Y	N	N	Y

F. M. Dos Santos et al. 2025	Y	Y	Y	Y	N	N	Y	Y
K. S. Teixeira et al. 2025	Y	Y	Y	Y	N	N	Y	Y
J. P. Escalera-Antezana et al. 2025	N	N	Y	Y	N	N	Y	N
E. Delatorre et al. 2025	Y	Y	N	Y	N	N	Y	Y
P. R. Martins-Filho et al. 2025	Y	Y	Y	Y	N	N	Y	Y
B. M. Bello-Rodríguez et al. 2025	Y	Y	N	N	Y	Y	N	N
S. T. S. de Lima et al. 2025	N	N	N	Y	N	N	Y	N
W. Silva-Caso et al. 2025a	Y	Y	N	Y	N	N	Y	N
F. C. de Melo Iani et al. 2025	Y	Y	N	Y	Y	Y	Y	N
E. A. Ramírez-García et al. 2025	Y	Y	Y	Y	N	N	Y	Y
M. P. Gomes Mourao et al. 2025	Y	Y	Y	Y	N	N	Y	Y
J. P. M. Nascimento et al. 2025	Y	Y	Y	Y	N	N	Y	Y
M. Sabalza et al. 2025	N	N	N	Y	Y	N	N	Y
W. Silva-Caso et al. 2025b	N	Y	Y	Y	N	N	Y	Y
A. C. Cruz et al. 2009	Y	Y	N	Y	N	N	N	Y
L. S. Catenacci et al. 2021	N	Y	N	Y	Y	Y	N	Y
N. Labiod et al. 2025	Y	Y	Y	Y	N	N	Y	Y
J. Gil-Mora et al. 2022	Y	Y	Y	Y	Y	Y	Y	Y
B. B. Salgado et al. 2021	Y	Y	Y	Y	Y	Y	Y	Y
O. Castro Peraza et al. 2025	Y	Y	Y	Y	N	N	Y	Y
J. P. Cola et al. 2025	Y	Y	Y	N	Y	Y	N	Y
E. B. Martins et al. 2025	Y	Y	Y	Y	Y	Y	N	Y

Question 1: Were the criteria for inclusion in the sample clearly defined?
Question 2: Were the study subjects and the setting described in detail?
Question 3: Was the exposure measured in a valid and reliable way?
Question 4: Were objective, standard criteria used for measurement of the condition?
Question 5: Were confounding factors identified?
Question 6: Were strategies to deal with confounding factors stated?
Question 7: Were the outcomes measured in a valid and reliable way?
Question 8: Was appropriate statistical analysis used?
N: No; Y: Yes.

A

Leave-one-out meta-analysis summary | Number of studies = 7
Random-effects model
Method: REML
SE adjustment: Knapp–Hartung
theta: Overall Freeman–Tukey's p

Omitted study	theta	[95% confidencial interval]		p value
J. W. LeDuc et al. 1981	0.449	0.027	0.871	0.057
K. J. Baisley et al. 1998	0.431	0.052	0.81	0.048
A. C. Bernardes-Terzian 2009	0.593	0.095	1.091	0.036
A. C. Cruz et al. 2009	0.558	0.041	1.074	0.052
L. S. Catenacci et al. 2021	0.601	0.11	1.092	0.033
B. B. Salgado et al. 2021	0.607	0.124	1.09	0.03
J. Gil-Mora et al. 2022	0.59	0.089	1.09	0.005
theta	0.547	0.131	0.963	0.003

B

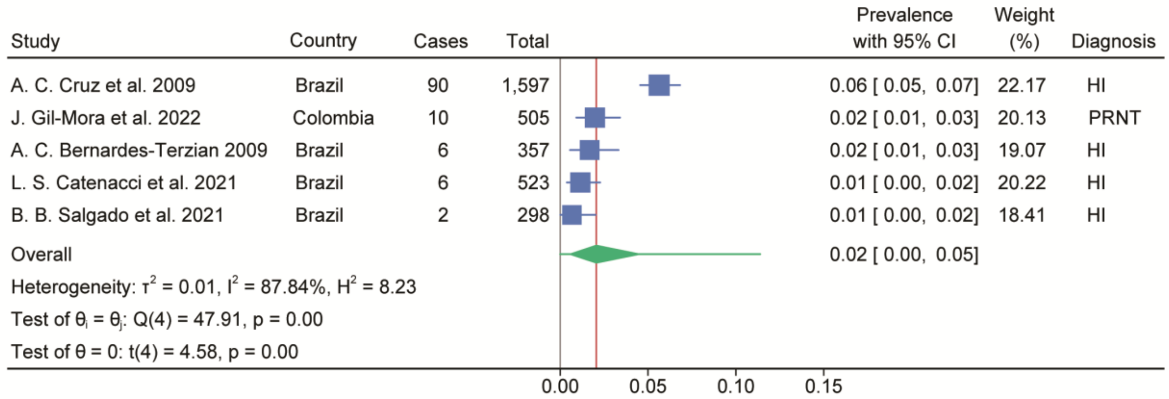

Figure S1. (A) Leave-one-out cross validation for the sensitivity analysis including studies with general population. (B) Estimated seroprevalence of anti-OROV antibody positivity in general population where outbreaks occurred excluding two pre-2000 studies. Blue symbol and line represent estimated prevalence of each study with 95% CI. Green symbol represents estimated prevalence with 95% CI. Green line represents 95% prediction interval.

A

```
  Effect-size label: Prevalence
        Effect size: _meta_es
          Std. err.: _meta_se

Random-effects meta-regression                    Number of obs  =          34
Method: REML                                      Residual heterogeneity:
SE adjustment: Knapp-Hartung                                  tau2 =      .184
                                                            I2 (%) =     99.68
                                                                H2 =    310.34
                                                     R-squared (%) =     24.43
                                                  Model F(4,29)    =      3.68
                                                  Prob > F         =    0.0153

    _meta_es   Coefficient  Std. err.      t    P>|t|     [95% conf. interval]

    ln_total    -.1832259   .0591291    -3.10   0.004    -.3041585   -.0622932

 starttime01
 before 2000    -.1285997   .2973795    -0.43   0.669     -.736809    .4796097
   2000~2009    -.7615069   .2994753    -2.54   0.017    -1.374003   -.1490111
   2010~2020    -.2149647   .2144598    -1.00   0.324    -.6535842    .2236547

       _cons     2.074322   .4490854     4.62   0.000     1.155839    2.992805

Test of residual homogeneity: Q_res = chi2(29) = 10895.07Prob > Q_res = 0.0000
```

B

```
  Effect-size label: Prevalence
        Effect size: _meta_es
          Std. err.: _meta_se

Random-effects meta-regression                    Number of obs  =          24
Method: REML                                      Residual heterogeneity:
SE adjustment: Knapp-Hartung                                  tau2 =     .3475
                                                            I2 (%) =     98.99
                                                                H2 =     99.23
                                                     R-squared (%) =     18.96
                                                  Model F(4,19)    =      2.28
                                                  Prob > F         =    0.0981

    _meta_es   Coefficient  Std. err.      t    P>|t|     [95% conf. interval]

    ln_total    -.2336064   .0794565    -2.94   0.008    -.3999108    -.067302

 starttime01
 before 2000     .5000776   .5120885     0.98   0.341    -.5717359    1.571891
   2000~2009     .4285115   .4751128     0.90   0.378    -.5659109    1.422934
   2010~2020     .1152415   .4866826     0.24   0.815    -.9033968     1.13388

       _cons     1.968971   .5783963     3.40   0.003     .7583733    3.179568

Test of residual homogeneity: Q_res = chi2(19) = 1399.71 Prob > Q_res = 0.0000
```

C

Leave-one-out meta-analysis summary   Number of studies = 34
Random-effects model
Method: REML
SE adjustment: Knapp–Hartung
theta: Overall Freeman–Tukey's p

Omitted study	theta	[95% confidencial interval]		p value
V. L. Carvalho et al. 2022	0.788	0.614	0.961	0
M. Gaillet et al. 2021	0.795	0.617	0.973	0
F. G. Naveca et al. 2018	0.8	0.621	0.98	0
A. P. Rosa et al. 1996	0.808	0.627	0.989	0
R. S. Azevedo et al. 2007	0.819	0.639	0.999	0
A. C. Bernardes-Terzian 2009	0.826	0.647	1.004	0
P. F. Vasconcelos et al. 1989	0.8	0.62	0.98	0
W. Silva-Caso et al. 2025a	0.778	0.61	0.946	0
J. W. LeDuc et al. 1981	0.797	0.618	0.977	0
H. B. Vasconcelos et al. 2009	0.81	0.629	0.991	0
J. Usuga et al. 2024	0.819	0.638	0.999	0
A. J. Benitez et al. 2024	0.764	0.608	0.919	0
C. Alva-Urcia et al. 2017	0.817	0.636	0.997	0
M. S. Bastos et al. 2012 & 2014	0.826	0.648	1.004	0
E. L. Wise et al. 2020	0.824	0.645	1.003	0
F. P. Pinheiro et al. 1976	0.801	0.621	0.981	0
W. Silva-Caso et al. 2019	0.809	0.628	0.99	0
W. Silva-Caso et al. 2025b	0.809	0.628	0.99	0
H. M. Moreira et al. 2024	0.818	0.637	0.999	0
J. Martins-Luna et al. 2020	0.802	0.622	0.983	0
M. P. Garcia et al. 2016	0.82	0.64	1	0
B. F. Cardoso et al. 2015	0.829	0.652	1.006	0
M. P. Gomes Mourao et al. 2025	0.822	0.643	1.002	0
K. A. Ciuoderis et al. 2022	0.815	0.634	0.996	0
K. S. Teixeira et al. 2025	0.796	0.617	0.974	0
L. H. M. Feitoza et al. 2025	0.795	0.617	0.974	0
J. P. M. Nascimento et al. 2025	0.817	0.636	0.998	0
S. T. S. de Lima et al. 2025	0.812	0.631	0.993	0
F. B. da Costa et al. 2025	0.824	0.644	1.003	0
F. M. Dos Santos et al. 2025	0.834	0.66	1.008	0
P. Escalera-Antezana et al. 2025	0.818	0.637	0.999	0
J. Z. Nodari et al. 2025	0.805	0.624	0.986	0
E. Delatorre et al. 2025	0.829	0.652	1.006	0
P. R. Martins-Filho et al. 2025	0.825	0.646	1.004	0
theta	0.81	0.635	0.986	0

D

Leave-one-out meta-analysis summary   Number of studies = 24
Random-effects model
Method: REML
SE adjustment: Knapp–Hartung
theta: Overall Freeman–Tukey's p

Omitted study	theta	[95% confidencial interval]		p value
A. J. Benitez et al. 2024	1.006	0.715	1.297	0
M. Gaillet et al. 2021	0.991	0.705	1.276	0
R. M. De Figueiredo 2004	1.035	0.745	1.325	0
H. B. Vasconcelos et al. 2009	0.98	0.7	1.26	0
M. Sabalza et al. 2025	1.022	0.73	1.315	0
H. B. Vasconcelos et al. 2009	0.975	0.698	1.252	0
H. B. Vasconcelos et al. 2009	0.982	0.701	1.264	0
V. L. Carvalho et al. 2022	1.004	0.713	1.294	0
M. S. Bastos et al. 2012 & 2014	1.053	0.77	1.336	0
V. G. da Costa et al. 2017	1.044	0.756	1.332	0
R. C. de Lima et al. 2024	1.035	0.744	1.326	0
P. F. Vasconcelos et al. 1989	0.981	0.7	1.262	0
R. S. Azevedo et al. 2007	1.009	0.717	1.301	0
R. S. Azevedo et al. 2007	1.006	0.715	1.297	0
F. P. Pinheiro et al. 1976	1.039	0.75	1.329	0
A. P. Rosa et al. 1996	0.962	0.695	1.229	0
S. R. Manock et al. 2009	1.058	0.777	1.338	0
K. A. Ciuoderis et al. 2022	1.043	0.755	1.331	0
M. P. Garcia et al. 2016	1.019	0.726	1.312	0
M. P. Mouraao et al. 2009	1.023	0.73	1.315	0
M. P. Gomes Mourao et al. 2025	1.047	0.761	1.333	0
P. V. Aguilar et al. 2011	1.029	0.737	1.321	0
D. M. Watts et al. 2022	1.055	0.773	1.337	0
B. M. Forshey et al. 2010	1.055	0.772	1.337	0
theta	1.019	0.739	1.298	0

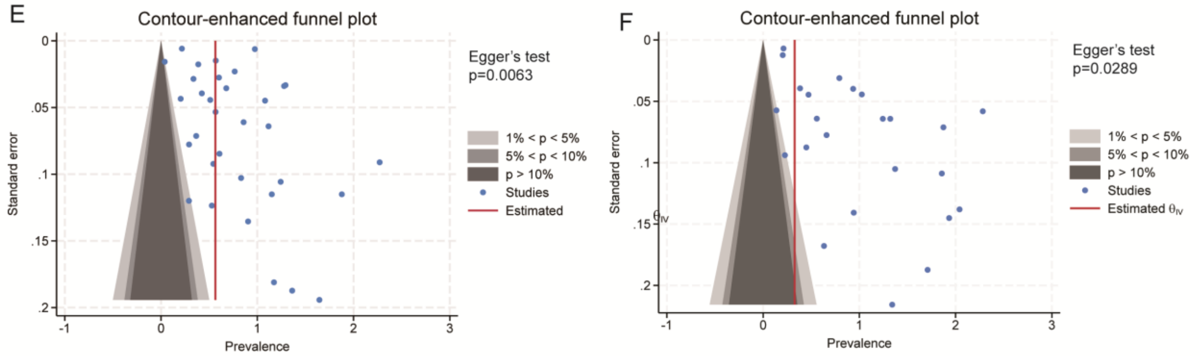

Figure S2. Meta-regression analysis of OROV prevalence estimates based on virus (A) and antibody (B) detection. Leave-one-out cross validation for the sensitivity analysis including studies with OROV infection based on virus (C) and antibody (D) detection in febrile patients. Assessment of publication bias in studies estimating the prevalence of OROV infection based on virus (E) and antibody (F) detection in febrile patients. "ln_total" denotes the natural logarithm of the study sample size (total number of

participants/samples). “starttime01” denotes the categorized study start period (before 2000; 2000–2009; 2010–2020; after 2020), and “after 2020” was used as the reference category.

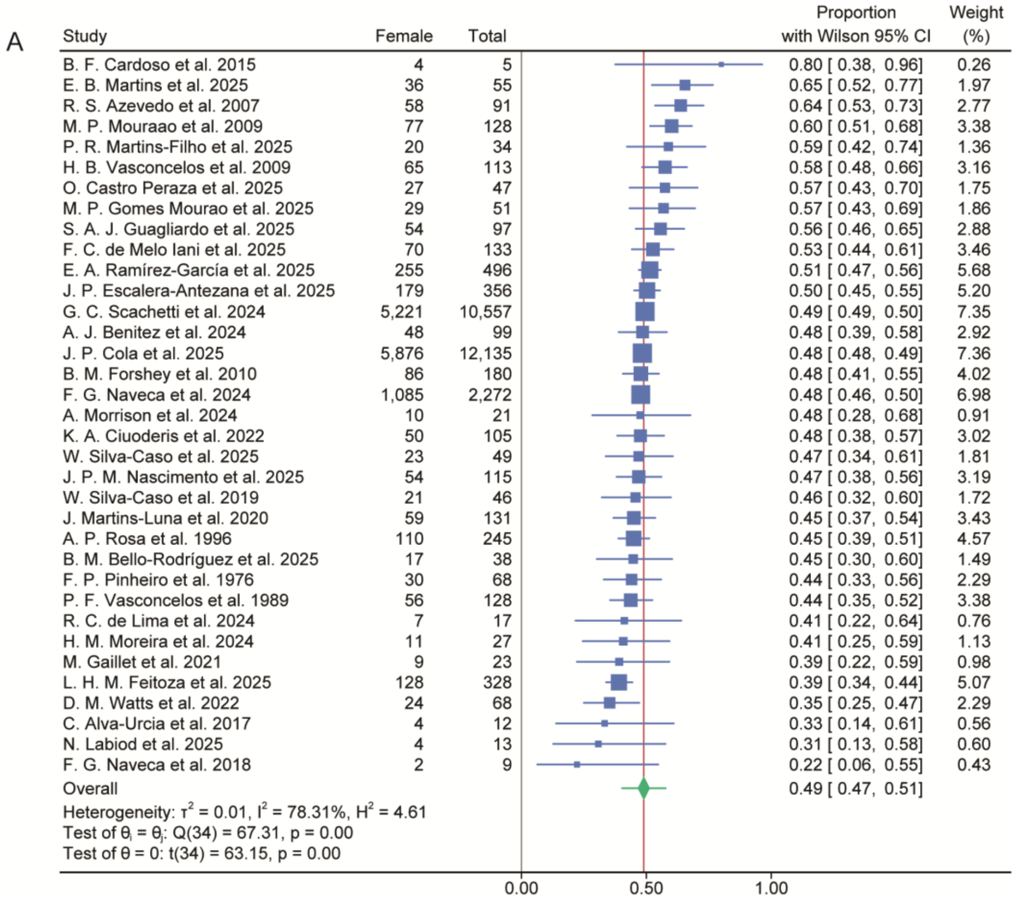

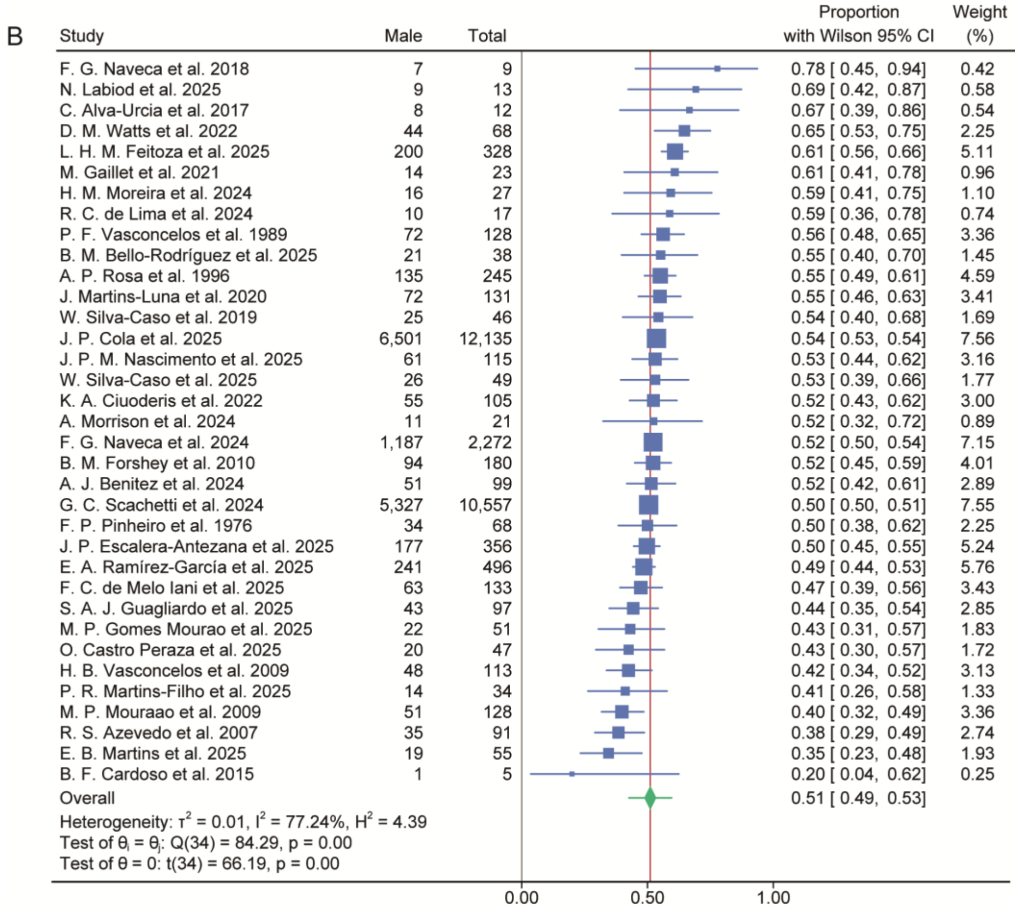

Figure S3. The frequency of women(A) and men(B) in OROV infected patients. Blue

symbol and line represent estimated prevalence of each study with 95% CI. Green symbol represents overall estimated prevalence with 95% CI. Green line represents 95% prediction interval.

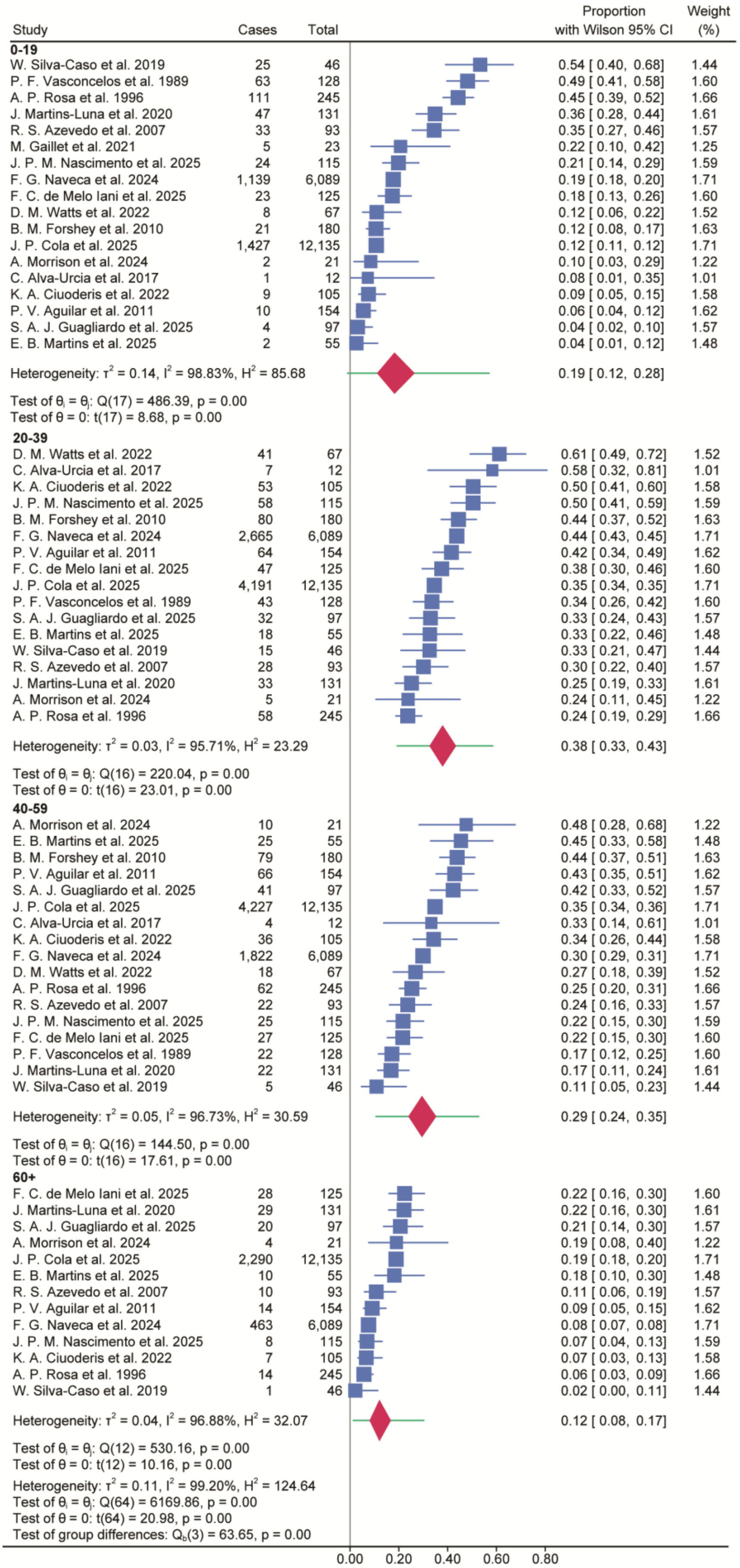

Figure S4. The frequency of different ages in OROV infected patients. Blue symbol and

line represent estimated prevalence of each study with 95% CI. Red symbol represents estimated prevalence of subgroup with 95% CI. Green line represents 95% prediction interval.

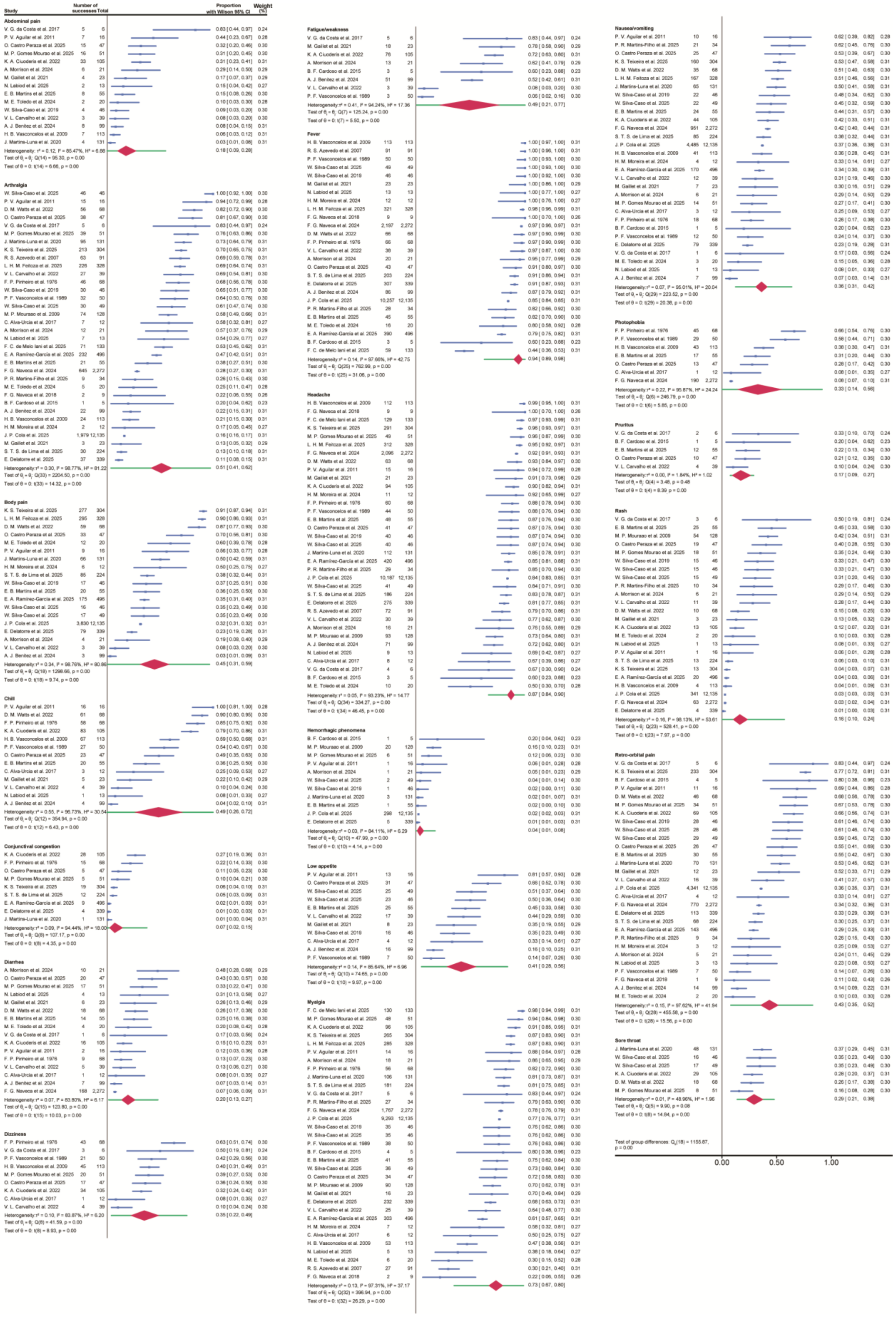

Figure S5. The frequency of various symptoms and signs in OROV infected patients. Blue symbol and line represent estimated prevalence of each study with 95% CI. Red

symbol represents estimated prevalence of subgroup with 95% CI. Green line represents 95% prediction interval.
